# Hacia rutas saludables: efecto de las etiquetas nutricionales en las conductas alimentarias en un comedor universitario

**DOI:** 10.1016/j.aprim.2021.102022

**Published:** 2021-03-31

**Authors:** Marta Cerezo-Prieto, Francisco Javier Frutos-Esteban

**Affiliations:** a Universidad de Salamanca, Salamanca, España; b Departamento de Sociología y Comunicación, Facultad de Ciencias Sociales, Universidad de Salamanca, Salamanca, España

**Keywords:** Comedor universitario, Arquitectura de elección, *Nudge*, Etiquetado nutricional, Promoción de la salud, Cambio de comportamiento, University canteen, Choice architecture, Nudge, Food labeling, Health promotion, Behavior change

## Abstract

**Objetivo:**

Evaluar la efectividad de incluir información nutricional y de propiedades de los alimentos en un comedor universitario de Salamanca (España), para promover las conductas alimentarias saludables.

**Diseño:**

Estudio experimental y correlacional transversal.

**Emplazamiento:**

Comedor universitario de Salamanca (España).

**Participantes:**

En el experimento se recogió información de la elección de 1.122 menús por parte de estudiantes universitarios. El cuestionario fue respondido por 48 estudiantes universitarios que participaron en el experimento.

**Medidores principales:**

Metodología mixta (experimento de campo y cuestionario en línea). La variable independiente fue la inclusión o no de información nutricional de los menús. Con el cuestionario se evaluó la actitud de los estudiantes sobre este tipo de herramientas.

**Resultados:**

El experimento muestra una mejora en la dieta de los estudiantes universitarios con la inclusión de elementos informativos que apelan a la elección más saludable, aumentando su consumo de fruta, verduras, legumbres, pescado y carne blanca.

Los encuestados mostraron un alto grado de receptividad de estas herramientas para la promoción de la salud. A pesar de esto, su autopercepción de mejoría de la dieta era más optimista que lo cuantificado en el experimento. Los estudiantes universitarios muestran un grado de aprobación muy alto frente a otras herramientas de promoción de alimentación saludable, especialmente aquellas de carácter educativo e informativo. Se comprobó que una preocupación mayor por la dieta estaba asociada con un mayor apoyo de estas herramientas.

**Conclusión:**

Existe una mejora en la alimentación de los estudiantes universitarios y una actitud positiva frente a herramientas de promoción de la salud, especialmente por parte de quienes tienen una autopercepción más saludable. Es necesario emplear nuevas herramientas basadas en las ciencias conductuales en la promoción de la salud, por parte de la industria privada y las entidades públicas.

## Abstract (en)

**Objective:**

To evaluate the effectiveness of including nutritional and food properties information in a university canteen in Salamanca (Spain) to promote healthy eating behaviours.

**Design:**

Experimental and correlational cross-sectional study.

**Location:**

University Dining Hall of Salamanca (Spain).

**Participants:**

In the experiment, information was collected on the choice of 1122 menus by university students. The questionnaire was answered by 48 university students who participated in the experiment.

**Main measurements:**

Mixed methodology (field experiment and online questionnaire). The independent variable was the inclusion or not of nutritional information from the menus. The questionnaire was used to evaluate the students’ attitude towards this type of tool.

**Results:**

The experiment shows an improvement in the diet of university students with the inclusion of information elements that appeal to the healthiest choice, increasing their consumption of fruit, vegetables, legumes, fish and white meat.

The students surveyed showed a high degree of receptivity to these health promotion tools. Despite this, their self-perception of dietary improvement was more optimistic than that quantified in the experiment. University students showed a very high degree of approval of other health promotion tools, especially those of an educational and informative nature. A greater concern for diet was associated with greater support for these tools.

**Conclusion:**

There is an improvement in the diet of university students and a positive attitude towards health promotion tools, especially by those with a healthier self-perception. There is a need for new tools based on behavioural sciences in health promotion by private industry and public entities.

## Introducción

España ocupa la séptima posición en la Unión Europea en cuanto a prevalencia de sobrepeso y obesidad en mayores de 15 años, comprendiendo un 61,6%^1^. Estas cifras son preocupantes desde una perspectiva de salud pública, ya que están asociadas con un mayor riesgo de enfermedades cardiovasculares y causa de mortalidad. Además de llevar una vida activa, hábitos como la alimentación son claves para reducir la obesidad y sus consecuencias en la salud.

Una de las etapas vitales que son claves para comenzar a tener hábitos individuales saludables es el periodo de la enseñanza superior, que se caracteriza en muchas ocasiones por el abandono del hogar y por el inicio de nuevos ritmos y actividades en el día a día^2^. En este sentido, la dieta de los estudiantes universitarios que abandonan el entorno familiar sufre también un cambio que será determinante para su salud en dicha etapa y en las siguientes. Los estudiantes que se alimentan en comedores universitarios donde se presenta la comida tipo *buffet* tienen un doble reto: elegir opciones más saludables y rechazar aquellas que no lo son. La falta de costumbre a la hora de tomar decisiones saludables, la ausencia de una autoridad como la familia y la presencia de otros estudiantes con estas limitaciones son factores que desencadenan que la alimentación saludable de este sector de la población se aleje de una dieta equilibrada. Los agentes políticos y sociales intentan fomentar hábitos alimentarios saludables introduciendo cambios en el conocimiento, las actitudes y las preferencias de los consumidores. Sin embargo, a pesar de los esfuerzos realizados, las medidas destinadas a educar e informar han tenido un éxito limitado.

En este contexto, las ciencias conductuales dan respuesta a la limitación conductual de la población. Los sesgos de decisión^3^ aparecen en cualquier elección, sea o no la más beneficiosa para el individuo, y también cuando elegimos qué comer. Estos sesgos demuestran que no somos, en todo momento, seres racionales y analíticos, sino que tomamos decisiones de manera impulsiva y rápida, limitando el procesamiento de la información que se nos presenta. Es por esto que los consumidores se dejan llevar por sus emociones, elecciones anteriores e incluso terceras personas también en el momento de elegir un alimento respecto a otro. Para comprender mejor el comportamiento de los individuos, las ciencias de la conducta proporcionan un enfoque novedoso para entender dos elementos: el comportamiento del consumidor en la toma de decisiones y el efecto del entorno en el que se toman^4^. Desde hace décadas, las ciencias de la conducta han investigado en el diseño e implementación de estrategias y herramientas que persuadan a los ciudadanos para que apuesten por una alimentación más saludable y responsable. Aplicables a una gran variedad de contextos relacionados con la salud pública, dichas estrategias han sido vehiculadas por entidades públicas y privadas mediante campañas en medios de comunicación, introduciendo patrones en la dieta como el plato de Harvard o legislando sobre la información nutricional disponible en los productos de consumo alimenticio. Por ejemplo, en la actualidad existen propuestas como el NutriScore, ya empleado en Francia y Bélgica, para facilitar la información más visual en los supermercados empleando un semáforo que indica los más y menos saludables. Actualmente, en España se está estudiando su implementación mediante un real decreto^5^. En este sentido, varios estudios han demostrado que tácticas como planificar el orden en que se muestran los productos, su color, el tamaño del plato o la inclusión del etiquetado nutricional pueden ayudar en la elección de alimentos más saludables^5^, ^6^, ^7^, ^8^, ^9^, ^10^, ^11^, ^12^, ^13^, ^14^.

Revisiones sistemáticas^6^, ^15^ han demostrado que se han tenido en cuenta muchos elementos físicos en experimentos dirigidos a individuos, incluida la colocación de productos, las señales ambientales (como el uso de símbolos de colores tipo semáforo para comunicar la información nutricional^7^) o el tamaño de las porciones^8^, ^16^ para dirigir el comportamiento hacia opciones más saludables.

En esta investigación se analiza el efecto de la inclusión de información en el momento de consumo. Se ha comprobado que, de este modo, la elección racional de los comensales se ve afectada por los juicios heurísticos^9^. Por ejemplo, se han observado cambios positivos en estudiantes en la selección de alimentos saludables al introducir «mensajes basados en beneficios» en un comedor^10^, ^11^. Otros estudios^12^, ^17^, ^18^ mostraron cómo la ubicación de la información, elementos gráficos llamativos o el contenido del mensaje son importantes para influir en la elección de alimentos saludables^19^, ^20^. En cuanto a la opinión o percepción de los comensales sobre la inclusión de estos elementos, se ha determinado^21^ que son considerados útiles, ya que amplían sus conocimientos sobre nutrición sin sentirse presionados en su elección. Dichas propuestas de intervención –también conocidas como *nudges*– fueron definidas en 2008 por Thaler y Sunstein^22^ como cualquier aspecto o elemento de la arquitectura de la elección que modifica la conducta de las personas de una manera predecible, sin prohibir ninguna opción, ni cambiar de forma significativa sus incentivos económicos. Para que dichos elementos puedan ser considerados *nudges* deben ser baratos y fáciles de evitar, es decir, no debe percibirse como una orden. Por ello, un buen ejemplo de *nudge* es colocar la fruta de forma muy visible para que pueda ser elegida frente a otras opciones de postre en el autoservicio de un comedor universitario. Sin embargo, y en ese mismo contexto, prohibir la comida basura no podría ser considerado un *nudge*, sino una medida coercitiva que aspira a corregir un comportamiento alimentario poco saludable. Aunque la Fundación del Español Urgente Fundéu-BBVA enumera otras alternativas válidas en castellano para evitar el anglicismo –como acicate, incentivo, estímulo o palanca, o los correlativos verbos animar, espolear o incitar–, lo cierto es que el término *nudge* se emplea comúnmente para referirse a la herramienta al servicio de políticas públicas, destinada a hacer más responsables socialmente los comportamientos individuales respecto a la salud o al medio ambiente. Por esa razón, en 2017, el Comité Económico y Social Europeo en su dictamen sobre cómo integrar los *nudges* en las políticas europeas los ubica como el quinto instrumento en manos de las autoridades para el fomento activo de las políticas públicas comunitarias^23^.

El objetivo del presente experimento es comprobar la efectividad de dichas herramientas en un comedor universitario. Un contexto social en el que las conductas alimentarias de los estudiantes de enseñanza superior deben elegir entre productos en el momento inmediatamente anterior a su ingesta, desapareciendo el tiempo que puede existir desde la compra hasta el consumo, como ocurriría en un supermercado.

## Método

La investigación se elaboró entre los meses de enero y febrero de 2020 en el comedor universitario del Colegio Mayor Fray Luis de León de la Universidad de Salamanca (España), midiendo el impacto de las etiquetas nutricionales en las conductas alimentarias de los estudiantes de enseñanza superior y, posteriormente, las actitudes sobre la inclusión de estas informaciones de una muestra de comensales. La investigación estuvo formada por dos estudios: uno de carácter experimental y otro cuantitativo, empleando un cuestionario.

### Experimento

El primer estudio muestra la elección de menú entre un grupo de estudiantes universitarios en un período control frente a otro con intervención experimental. Antes del experimento, se obtuvo el correspondiente consentimiento informado por parte del Colegio Mayor, la empresa de *catering* y los estudiantes.

El hecho de analizar la elección de menús en un comedor de un Colegio Mayor proporcionaba un número de estudiantes estable en el tiempo en que, además, acudían a diario al comedor por ser colegiales de la residencia. El número de estudiantes matriculados en el Colegio Mayor en el momento en que se realizó el experimento era de 89. En los 14 días de análisis, la media de estudiantes que asistieron al comedor a diario fue de 73 (desviación típica [DT] = 9,8), todos ellos eran colegiales. Durante los 14 días, la media de hombres que acudió al comedor era de 43,46% y de mujeres de 56,54% (DT = 5,4). El menú del comedor ofrecía un primer plato, un segundo plato con o sin guarnición (compuesta siempre por patatas fritas o lechuga) y un postre. Se ofrecían tres opciones de cada plato. La intervención consistió en colocar de manera gráfica información nutricional de cada comida ofertada. Para elaborar la investigación longitudinal, se hizo un análisis previo a la colocación de las etiquetas (durante siete días) y otro durante su inclusión (siete días), estando presente un codificador para la toma de información.

### Grupo control

En primer lugar, se llevó a cabo el análisis del grupo control, sin intervención, codificando el sexo del comensal y la elección de platos que hacía. En total se codificaron 506 menús, con una media de 72 al día (DT = 10). La muestra de estudiantes estaba compuesta por un 42,3% de hombres y un 57,7% de mujeres.

### Grupo experimental

En segundo lugar, para la obtención de la información nutricional de los menús, se contactó con la empresa responsable del *catering* del Colegio Mayor, que nos proporcionó todos los platos que ofrecía y las informaciones por una ración. A continuación, se elaboraron carteles con información nutricional en una escala de colores tipo semáforo de cada plato (verde, amarillo o rojo). Los puntos de corte para establecer los colores del semáforo se basaron en los criterios de la *Food Standards Agency* del Reino Unido^24^, calculados en relación con una ración habitual de consumo (tabla 1).

**Tabla 1:** Puntos de corte para el etiquetado semáforo, expresados por ración habitual de consumo

Nutriente	Bajo (verde) < 7,5% CDO	Medio (amarillo)	Alto (rojo) > 20% CDO
Energía (kcal)	≤ 150	150-400	≥ 400
Grasa (g)	≤ 5,25	5,25-14	≥ 14
Grasa saturada (g)	≤ 1,5	1,5-4	≥ 4
Azúcar (g)	≤ 4,5	4,5-12	≥ 12
Sal (g)	≤ 0,45	0,45-1,2	≥ 1,2

**Figura 1:**
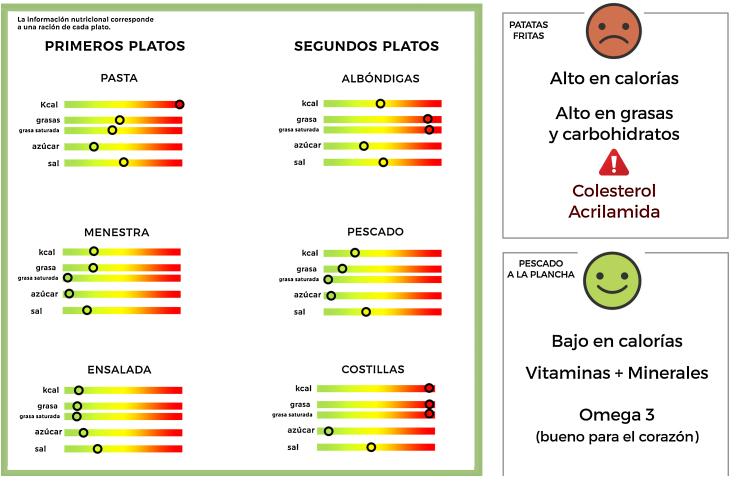
Ejemplo de cartel con información nutricional del menú del día y etiquetas en las bandejas de patatas fritas y pescado a la plancha.

Además del cartel, dentro del comedor y delante de cada bandeja, se colocó una etiqueta informativa con propiedades nutricionales de cada plato, acompañada de una pequeña cara triste, neutra o sonriente y roja, amarilla o verde, dependiendo de lo saludable que fuera cada plato. Así, por ejemplo, las patatas fritas como guarnición presentaban una etiqueta con una cara triste roja o el pescado a la plancha una cara contenta verde (fig. 1).

Una vez diseñadas ambas intervenciones, se colocaron en sus lugares y se realizó el mismo estudio que en el grupo control durante siete días para así cuantificar las diferencias de elección de los alimentos. En este grupo experimental se codificaron 595 menús, con una media de 74 al día (DT = 8). La muestra de estudiantes estaba compuesta por un 43,9% de hombres y un 56,1% de mujeres.

Una vez recolectados los datos, se trasladaron al *software* SPSS versión 25 (IBM Corp, Armonk, NY), donde se hizo el análisis estadístico. Se descartaron de la muestra los estudiantes extranjeros (ya que presentaban patrones de consumo diferentes a los nacionales) y aquellos estudiantes que solo escogían un plato del menú.

### Cuestionario

Para complementar el estudio anterior, y una vez finalizado, se diseñó un cuestionario piloto que fue distribuido mediante una encuesta en línea a los colegiales que a diario comían en el comedor. Esta encuesta estuvo disponible durante una semana, dos semanas después de finalizar el experimento, y estaba distribuida en los siguientes bloques de preguntas: sociodemográficas, sobre las intervenciones, sobre la aprobación de otras herramientas de fomento de una alimentación saludable por parte de las administraciones públicas y sobre la autopercepción de sus hábitos alimentarios. Este método ha sido empleado anteriormente para estudiar el grado de aceptación de políticas públicas en salud (*nudges*) y su relación con el estilo de vida saludable, concluyendo que existe una relación positiva entre ambas^25^, ^26^, ^27^.

La encuesta fue contestada por 48 estudiantes entre 19 y 24 años (M = 20), de los cuales, el 70,83% eran mujeres y el 29,17% hombres.

**Figure:**
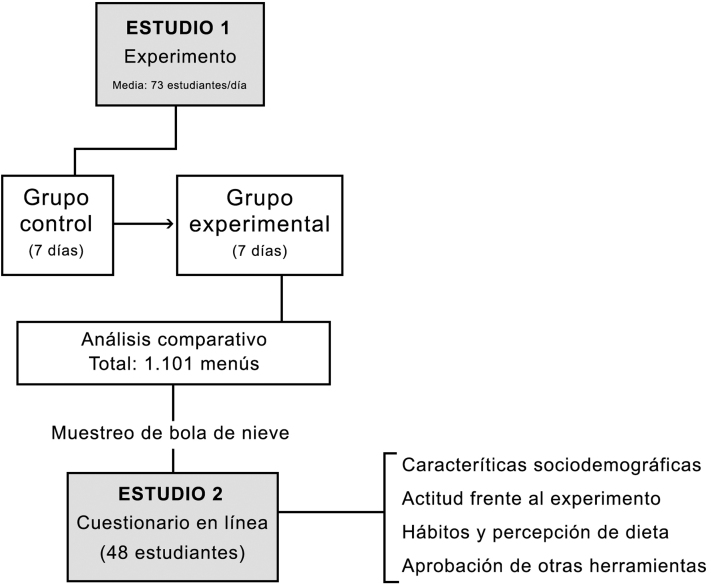
**Esquema general del estudio**. Estudio experimental y correlacional a una muestra de población universitaria en el contexto de un comedor de un Colegio Mayor de Universidad.

## Resultados

### Experimento

En primer lugar, se analizaron los dos grupos del experimento de manera individual, con el fin de conocer si existían relaciones entre el sexo de los estudiantes y la elección de platos. Así, en el grupo control se encontraron resultados estadísticamente significativos entre el sexo y el segundo plato (χ^2^ [5, n = 505] = 19,92, p < 0,001), siendo los hombres quienes comían más carne roja (43,5 vs. 38,8% de mujeres), más carne blanca (29,4 vs. 20,3% de mujeres) y las mujeres quienes comían más pescado (12 vs. 3,3%). También se encontraron relaciones significativas con la guarnición (χ^2^ [3, n = 505] = 48,3, p < 0,001), siendo los hombres quienes comían más patatas fritas (78 vs. 56,5% de mujeres), más ensalada (13,1 vs. 8,2% de mujeres) y las mujeres quienes preferían no comer guarnición (35,3 vs. 8,9% de hombres). Por último, en relación con el postre (χ^2^ [4, n = 505] = 9,**7**, p < 0,05), se encontró que las mujeres elegían más fruta (36,3 vs. 28% de hombres), menos yogur (42,1 vs. 53,7% de hombres) y eran más numerosas a la hora de no tomar postre (15,8 vs. 10,3%).

En el grupo experimental se encontraron resultados estadísticamente significativos entre el sexo y el segundo plato (χ^2^ [5, n = 595] = 27,5, p < 0,001), siendo los hombres quienes comían más carne roja (26,8 vs. 21,3% de mujeres), más carne blanca (33,7 vs. 26%) y las mujeres quienes consumían más pescado (21 vs. 10,3%), al igual que en el grupo control. También se encontraron relaciones significativas con la elección de la guarnición (χ^2^ [3, n = 595] = 81, p < 0,001), siendo los hombres quienes comían más patatas fritas (74,7 vs. 41,3% de mujeres) y las mujeres quienes preferían no comer guarnición (44,9 vs. 12,3% de hombres). El porcentaje de ensaladas aumentó en el caso de las mujeres respecto a los hombres (13,5 vs. 13%).

Tras este análisis, se procedió a recodificar las variables para elaborar una comparativa entre el grupo control y el experimental. El grupo control estaba compuesto por un 42,3% de hombres y un 57,5% de mujeres (n = 506), mientras el grupo experimental por un 43,9% de hombres y un 56,1% de mujeres (n = 595). Durante el análisis estadístico se calcularon las medias del porcentaje de consumo de varias categorías de alimentos, distinguiendo si era antes o durante la intervención.

**Figura 2:**
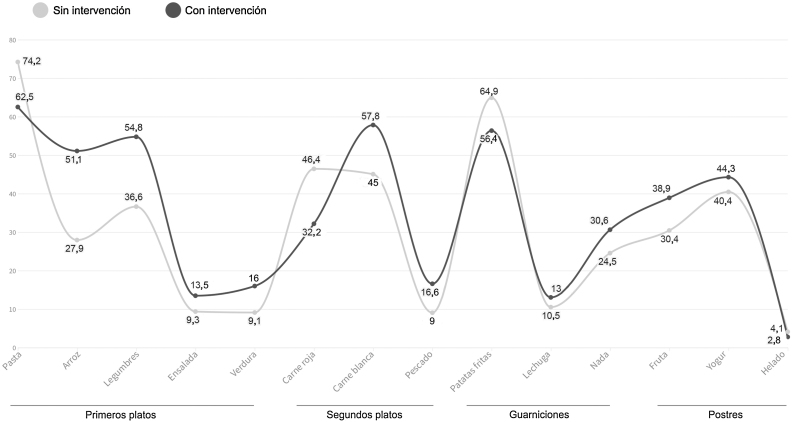
Consumo de platos antes y durante la intervención experimental (%).

En la figura 2 se muestra el porcentaje de los estudiantes que consumía cada tipo de alimento, antes y durante las intervenciones.

De estas cifras se pudo concluir que la dieta de los estudiantes mejoró considerablemente, aumentando el consumo de legumbres, ensalada, carne blanca, pescado, fruta y yogur. Existían diferencias estadísticamente significativas en todos los platos: guarnición (χ^2^ [4, n = 1.101] = 12,3, p < 0,05), primer plato (χ^2^ [6, n = 1.101] = 125, p < 0,000), segundo plato (χ^2^ [6, n = 1.101] = 69,5, p < 0,000) y postre (χ^2^ [4, n = 1.101] = 26,7, p < 0,000).

### Cuestionario

Todos los encuestados eran estudiantes de grado que acudían diariamente al comedor, lo que nos aseguraba que habían participado en el estudio anterior. En un primer bloque de preguntas se consultó sobre las intervenciones del comedor. A la evaluación del grado de utilidad de las dos intervenciones, en una escala del 0 al 10, los carteles informativos fueron puntuados con un 4,9 (DT 2,8) y las etiquetas con caras con un 6,8 (DT 2,3). No existían diferencias significativas entre la utilidad percibida y el sexo (t [46] = -0,474, p = 0,638) (t [46] = -0,351, p = 0,727]; sin embargo, las mujeres las evaluaban como más útiles.

A la pregunta sobre la influencia de las intervenciones en la elección de alimentos, el 32,4% reconocía que les ayudaron a comer menos patatas fritas, el 14,7% a comer más fruta, el 13,7% a comer más legumbres, el 12,8% a comer menos pasta, el 12,8% a comer más pescado, el 5,9% comer más ensalada y el 2,9% a comer menos carne.

Al comparar estos porcentajes con la diferencia porcentual en la elección de platos del primer estudio, se comprobó que los encuestados tenían una autopercepción más optimista de su cambio de dieta en el caso de la pasta, ensalada, pescado, patatas y fruta, ya que, a pesar de que se mejoró la dieta, no había sido en tanto porcentaje como manifestaban.

Sobre la percepción de las intervenciones se obtuvieron resultados que alientan a su permanencia y utilidad (tabla 2).

**Tabla 2:** Respuestas a la pregunta: «¿Las intervenciones te han ayudado a elegir qué comer? Selecciona las opciones con las que te sientas identificado/a»

Afirmaciones con las que se sienten identificados/as	%
Las caras tristes les hacían sentir culpables de sus malos hábitos	28
Les han aportado información que desconocían anteriormente sobre las propiedades de los alimentos	25,6
Se tomaron como reto personal elegir las opciones más saludables	24,4
Manifestaron sentirse manipulados con estas intervenciones	0
Los carteles y las etiquetas deberían estar presentes de manera permanente	79,2
Reconocieron volver a comer como lo hacían anteriormente en el momento en que el *nudge* se eliminó	64,6

Cabe señalar que los estudiantes que se mostraron más positivos frente a los dos tipos de intervenciones son quienes declaraban tener una dieta más sana que el resto (p < 0,005), y quienes se preocupaban por su cuerpo en verano (p < 0,05). Por otro lado, el 87,1% de encuestados que reconocieron volver a su dieta anterior cuando se eliminaron las etiquetas, estarían en favor de su permanencia, por lo que podría ser una buena medida para frenar el regreso a hábitos menos saludables.

En un segundo bloque de preguntas, se solicitó que contestaran si aprobarían o no 14 medidas relacionadas con la promoción de alimentación saludable si las propusiera el gobierno. Estas medidas han sido adaptadas de Kwon et al., 2019^28^.

La media de aprobación era de 12,1 medidas (DT = 2,8). En la tabla 3 se muestra el porcentaje de apoyo de cada propuesta.

**Tabla 3:** Listado de medidas y porcentaje de apoyo

Medidas	%
El Gobierno exige una etiqueta calórica en cadenas de restaurantes	100
Para reducir la obesidad infantil, el Gobierno emplea una campaña educativa que consiste en ofrecer información a los padres para ayudarles a tomar decisiones más saludables para sus hijos	100
El Gobierno exige incluir etiquetas en productos que tienen niveles de sal inusuales como: «En este producto se han encontrado altos niveles de sal, lo que puede ser perjudicial para su salud»	97,9
El Gobierno exige un sistema de «semáforo de alimentos», con el que la comida sana se venderá con un pequeño icono verde, la comida no saludable con un icono rojo y las que no son especialmente sanas o no saludables con un icono amarillo	87,5
Una ley que exija a los fabricantes un límite máximo permitido en los niveles de sal o azúcar en los alimentos envasados, modificando así la fórmula de los alimentos para que sean más saludables	87,5
Una ley que obliga a las grandes cadenas de alimentación a poner la comida más sana en los lugares más visibles para los clientes (a la altura de los ojos, cerca de las cajas)	77,1
El Gobierno exige a las salas de cine poner, antes de las películas, mensajes educativos contra el tabaco y el sobrepeso	68,8
Para detener el aumento de obesidad, el Gobierno exige a los supermercados tener las zonas de los cajeros sin dulces	62,5
El Gobierno elimina en televisión la publicidad sobre alimentos ultraprocesados en horario infantil (galletas, bollería, precocinados)	62,5
El Gobierno propone una ley que restringe el número de restaurantes de comida rápida cerca de los colegios	60,4
Por razones de salud y protección del medio ambiente, el Gobierno exige a los comedores de las instituciones públicas (comedores universitarios, colegios públicos, administraciones) a tener un día sin carne a la semana	60,4
Un impuesto sobre las bebidas azucaradas o alimentos con alto contenido de azúcar (lo que incrementaría su precio en el mercado)	52,1
El Gobierno obliga a incluir publicidad subliminal para que la gente no fume o coma en exceso en las salas de cine (por ejemplo, anuncios que pasan tan rápidamente que la gente no los percibe conscientemente)	50
El Gobierno prohíbe comercializar bebidas azucaradas para niños	41,7

Las herramientas con resultados más positivos son las relacionadas con campañas educativas y el aumento y mejora de la información nutricional en los productos. Las menos apoyadas son aquellas que requieren una subida del precio de los productos o que restringen la comercialización de alimentos menos saludables. Se elaboró un índice de apoyo de medidas, cuya consistencia interna se evaluó mediante el coeficiente alfa de Cronbach. El valor obtenido fue de 0,8, lo que indica una adecuada fiabilidad del ítem. Este índice se correlacionó con otras variables del cuestionario, dando una relación significativa positiva con aquellos que puntúan como más útiles las etiquetas en el comedor (p < 0,01). También existía una relación positiva entre el mayor apoyo de estas medidas y quienes tienen hábitos más saludables. Así, quienes consideran que comen más sano que el resto de sus amigos (p < 0,05), que comen suficientes frutas y verduras (p < 0,05) y que comen más pescado (p < 0,05) aprobarían más cantidad de *nudges*.

## Discusión

Los resultados de este estudio sugieren que los *nudges* relacionados con las etiquetas nutricionales pueden ayudar a elegir alimentos más saludables en el contexto de un comedor universitario. Sin embargo, dado el pequeño tamaño de la muestra, existen limitaciones en estos hallazgos.

En primer lugar, este estudio se centra únicamente en la población universitaria, por lo que generalizar requiere que los resultados se repitan en un entorno no universitario. Asimismo, a pesar de que la población universitaria presenta una alimentación inadecuada, y dada la estipulación previa de los menús por parte de la empresa de *catering*, no resulta viable inferir los platos elegidos a la población de estudiantes que vive con sus familias o con compañeros de piso. Además, las muestras analizadas en las dos fases del experimento no estaban conformadas por las mismas personas en su totalidad, ya que algunos días preferían comer fuera del Colegio Mayor. Por otra parte, la muestra de sujetos que contestó la encuesta estaba conformada por un porcentaje de mujeres mayor que el que participó en el experimento, por lo que la percepción de los hombres estaba infrarrepresentada. Existen resultados heterogéneos respecto a la efectividad de los *nudges* en el largo plazo. Algunos estudios^29^, ^30^ han determinado que, a largo plazo, los *nudges* pierden efectividad por el hecho de que los usuarios se acostumbran al mismo y retoman sus hábitos anteriores, mientras que otros sí han encontrado una eficacia de estas herramientas en el largo plazo. Por tanto, este experimento debería ampliar el tiempo de estudio del grupo experimental, con el fin de evaluar la evolución en el largo plazo de las conductas alimentarias.

Sin embargo, este estudio aborda por vez primera en España la eficacia de las etiquetas nutricionales en un grupo de población, un paso indispensable para la visibilización del empleo de *nudges* en el país. Si bien estos resultados no son necesariamente representativos de todos los estudiantes universitarios, los hallazgos sugieren una efectividad potencial para algunas intervenciones asequibles y de baja interferencia para fomentar una alimentación saludable.

En particular, los resultados del estudio muestran que el empleo de información nutricional en comedores universitarios es eficaz para mejorar la calidad de la dieta, reduciendo el consumo de patatas fritas, carne roja, pasta y helado. El experimento no afectó más a hombres o mujeres, ya que en las dos situaciones los hombres consumían más carne y patatas que las mujeres, siendo estas quienes preferían más el pescado o no elegir guarnición.

A pesar de esta mejora de la dieta, en el segundo estudio se comprobó que el grupo de encuestados se mostró muy optimista en su cambio de actitud al elegir los platos, ya que la percepción que tenían de mejora de su alimentación no coincidía con las diferencias porcentuales que aparecían en el experimento.

De manera más general, este estudio confirma que es posible diseñar *nudges* para promover un comportamiento deseado (por ejemplo, mejorar la calidad de la dieta), sin un esfuerzo consciente, y aun así dejar que los consumidores tengan la libertad de tomar una decisión^31^. Teniendo en cuenta que una regulación normativa estricta a menudo no es eficaz para cambiar comportamientos hacia una dieta saludable^9^, el empleo de *nudges* en entornos específicos podría ser un método eficaz.

El segundo estudio a una muestra de comensales ha revelado un gran apoyo de estos sobre las intervenciones experimentales, tanto de los carteles con información nutricional como de las etiquetas individuales en cada plato, valorándose como útiles y declarando que deberían quedarse de manera permanente. Así, los encuestados reconocían sentirse culpables cuando optaban por un plato menos saludable y veían una cara triste en el mismo y haber ampliado sus conocimientos nutricionales sobre la oferta del comedor.

Las etiquetas con expresiones de caras tuvieron mejor acogida, ya que los mensajes que apelan a sentimientos como la culpa o que muestran alegría ante una buena elección eran más persuasivos que los que solo ofrecían información nutricional objetiva. Aquellos que manifestaron más utilidad de las etiquetas, eran quienes autopercibían su dieta como mejor que la del resto y se preocupaban más por su físico, de lo que se podía concluir que aquellos que seguían unos hábitos nutricionales más saludables eran más proactivos en la aceptación de estas intervenciones en su día a día.

Cuando se presentaron varios supuestos de aplicación de varias herramientas y medidas para mejorar la dieta de la población, se encontró un gran apoyo general, siendo las medidas de carácter educativo e informativo las que obtuvieron mayor aprobación que las que implicaban un aumento en el precio de los productos o eran más restrictivas. De cara a futuros diseños por parte de las administraciones públicas y las empresas privadas dedicadas a la nutrición, este estudio puede marcar varias pautas de las que partir. En primer lugar, respecto al diseño de información nutricional, los encuestados han declarado que los mensajes que incluyen expresiones o sentimientos han sido más efectivos que la información calórica. En este sentido, para campañas centradas en jóvenes o niños, se podrían plantear mensajes más emocionales relacionando el bienestar con la buena alimentación, y viceversa.

En segundo lugar, dado que la autopercepción de la alimentación es muy optimista, en comparación con las pautas alimenticias reales, y que quienes declaran comer más saludable también apoyarían otras medidas de promoción de la salud, se debe aclarar y promover, mediante campañas de comunicación y educación, en qué consiste una alimentación saludable (para que la autopercepción sea realista). Además, se deben aunar esfuerzos para hacer llegar mensajes más persuasivos a los grupos de población cuya alimentación es menos saludable y quienes la retoman cuando los mensajes nutricionales desaparecen. Consiguiendo esto, además, se obtendrá un mayor porcentaje de población que apoye otras propuestas públicas en favor de la salud.

**Lo conocido sobre el tema**

• Las ciencias de la conducta son grandes aliadas en el diseño de herramientas que mejoran el estilo de vida y la salud de la ciudadanía, como la alimentación. La mejora de estos hábitos en la etapa universitaria es clave para establecer unas pautas de comportamiento a largo plazo.
• Es necesario conocer los hábitos de la población para así diseñar métodos de promoción de la salud ajustados a sus características y necesidades.
• La población española dista de llevar una vida plenamente saludable y su dieta se ve afectada por patrones occidentales menos saludables.

**Qué aporta este estudio**

• Primer estudio realizado en España sobre la aplicación de *nudges* para mejorar la alimentación.
• Se constata la efectividad de las etiquetas con información nutricional para elegir alimentos más saludables en la población universitaria.
• La población universitaria se presenta muy en favor de estas nuevas herramientas como políticas públicas de promoción de la salud.

## Financiación

Esta investigación ha sido posible gracias al apoyo de la Junta de Castilla y León y el Fondo Social Europeo, a través de la ayuda destinada a financiar la contratación predoctoral de personal investigador (EDU/574/2018).

## Conflicto de intereses

Los autores declaran no tener ningún conflicto de intereses.

## References

[1] OECD (2019). The Heavy Burden of Obesity: The economics of prevention.

[2] The Keystone Center (2006). The Keystone forum on away-from-home foods: opportunities for preventing weight gain and obesity.

[3] Kahneman D. (2011). Thinking fast and slow.

[4] Abellán J.M., Jiménez-Gómez D. (2020). Economía del comportamiento para mejorar estilos de vida y reducir factores de riesgo. Gac Sanit..

[5] Ministerio de Consumo (2020). Proyecto de real decreto relativo a la utilización voluntaria del logotipo nutricional «Nutri-Score» en los productos alimenticios.

[6] Vecchio R., Cavallo C. (2019). Increasing healthy food choices through nudges: A systematic review. Food Qual Prefer..

[7] Thorndike A.N., Riis J., Sonnenberg L.M., Levy D.E. (2014). Traffic-light labels choice architecture: Promoting healthy food choices. Am J Prev Med..

[8] Hansen P.G., Skov L.R., Jespersen A.M., Skov K.L., Schmidt K. (2016). Apples versus brownies: A field experiment in rearranging conference snacking buffets to reduce short-term energy intake. J Foodserv Bus Res..

[9] Leng G., Adan R.A.H., Belot M., Brunstrom J.M., De Graaf K., Dickson S.L. (2017). The determinants of food choice. Proc Nutr Soc..

[10] Peterson S., Duncan D.P., Null D.B., Roth S.L., Gill L. (2010). Positive changes in perceptions and selections of healthful foods by college students after a short-term point-of-selection intervention at a dining hall. J Am Coll Health..

[11] Deliens T., Van Crombruggen R., Verbruggen S., De Bourdeaudhuij I., Deforche B., Clarys P. (2016). Dietary interventions among university students: A systematic review. Appetite..

[12] Buscher L.A., Martin K.A., Crocker S. (2001). Point-of-purchase messages framed in terms of cost, convenience, taste, and energy improve healthful snack selection in a college foodservice setting. J Am Diet Assoc..

[13] Boehm R., Read M., Henderson K., Schwartz M. (2020). Removing competitive foods v. nudging and marketing school meals: A pilot study in high-school cafeterias. Public Health Nutr..

[14] Dubber P.M., Johnson W.G., Schlundt D.G., Montague N.W. (1984). The influence of caloric information on cafeteria food choices. J Appl Behav Anal..

[15] Marcano-Olivier M.I., Horne P.J., Viktor S., Erjavec M. (2020). Using Nudges to Promote Healthy Food Choices in the School Dining Room: A Systematic Review of Previous Investigations. J Sch Health..

[16] Geier A.B., Rozin G., Doros P. (2006). Unit bias: A new heuristic that helps explain the effect of portion size on food intake. Psychol Sci..

[17] Freedman M.R., Connors R. (2010). Point-of-purchase nutrition information influences food-purchasing behaviors of college students: A pilot study. J Am Diet Assoc..

[18] Sogari G., Li J., Lefebvre M., Menozzi D., Pellegrini N., Cirelli M. (2019). The Influence of Health Messages in Nudging Consumption of Whole Grain Pasta. Nutrients..

[19] Cioffi C.E., Levitsky D.A., Pacanowski C.R., Bertz F. (2015). A nudge in a healthy direction The effect of nutrition labels on food purchasing behaviors in university dining facilities. Appetite..

[20] Schindler-Ruwisch J., Gordon M. (2020). Nudging healthy college dining hall choices using behavioral economics. J Am Coll Health..

[21] Vermote M., Nys J., Versele V., D’Hondt E., Deforche B., Clarys P. (2020). The effect of nudges aligned with the renewed Flemish Food Triangle on the purchase of fresh fruits: An on-campus restaurant experiment. Appetite..

[22] Thaler R.H., Sunstein C.R. (2008). Nudge: improving decisions about health, wealth and happiness.

[23] Libaert T. (2017). Dictamen del Comité Económico y Social Europeo sobre Integrar los *nudges* en las políticas europeas. Diario Oficial de la Unión Europea.

[24] Food Standards Agency (2007). Traffic light signpost labeling Technical Guidance.

[25] Cerezo-Prieto M., Frutos-Esteban F.J. (2020). Impacto del estilo de vida de los estudiantes universitarios en la promoción de políticas públicas en salud. El caso de los *nudges*. Rev Esp Salud Publica..

[26] Sunstein C.R., Reisch L.A., Kaiser M. (2019). Trusting nudges? Lessons from an international survey. J Eur Public Policy..

[27] Reisch L.A., Sunstein C.R. (2016). Do Europeans like nudges?. Judgm Decis Mak..

[28] Kwon J., Cameron A.J., Hammond D., White C.M., Vanderlee L., Bhawra J. (2019). A multi-country survey of public support for food policies to promote healthy diets: Findings from the International Food Policy Study. BMC Public Health..

[29] Lin Y., Osman M., Ashcroft R. (2017). Nudge: concept, effectiveness, and ethics. Basic Appl Soc Psychol..

[30] Sunstein C.R. (2017). Nudges That Fail. Behav Publ Pol..

[31] Van Kleef E., Van Trijp H.C.M. (2018). Methods in Consumer Research.

